# Stability of cytoplasmic nanoviscosity during cell cycle of HeLa cells synchronized with Aphidicolin

**DOI:** 10.1038/s41598-019-52758-6

**Published:** 2019-11-11

**Authors:** Krzysztof Szczepański, Karina Kwapiszewska, Robert Hołyst

**Affiliations:** Institute of Physical Chemistry, Polish Academy of Sciences, Kasprzaka 44/52, 01-224 Warsaw, Poland

**Keywords:** Nanoscale biophysics, Biological physics

## Abstract

Nanoviscosity of the cytoplasm is a key factor affecting diffusion of biomolecules and – as a consequence – rates of biochemical reactions in a cell. Nanoviscosity is an outcome of variable chemical and structural factors, which can temporarily change with cell-cycle associated changes of intracellular architecture. Thus, the question arises, whether rates of biochemical reactions depend on the point of cell cycle. In this paper we address this topic by constant observation of nanoviscosity of HeLa cells cytoplasm during S, G2 and G1 phases after Aphidicolin synchronization. For this purpose we measured diffusion rates of EGFP molecules using fluorescence correlation spectroscopy (FCS). To our surprise, a counter-intuitive stability of cytoplasmic viscosity was observed during the cell cycle. Our results hint at possible existence of robust mechanism maintaining stable physiological viscosity of the cytoplasm, despite huge structural changes during cell cycle.

## Introduction

Nanoviscosity of cytoplasm is defined as viscosity experienced by objects of nanometer-size^1^. Mobility and diffusion of molecules depend strongly on molecular crowding^2–4^. As a result, nanoviscosity affects all diffusion-based interactions in living cell, including biosynthesis, signalling pathways or even active transport^5^. Both mobility and diffusion of proteins and other components of cell cytoplasm have been measured in several studies^4,6,7^. Although there is information hinting on possible changes of viscosity of cytoplasm during the cell cycle^8–10^, no work focused its attention on those fluctuations.

The cell cycle is a universal process during which, through a series of cellular events, single cell gives rise to two daughter individuals. The cell cycle of somatic cells is commonly divided into two periods: interphase and mitosis. During the former one first and second gap phases can be distinguished (G1 and G2 respectively) with DNA synthesis (S phase) in-between them. During the G1 phase cells prepare for DNA synthesis. In S phase cellular DNA is doubled. The G2 phase serves as control point during which DNA replication is checked. Also at this point cells prepare for subsequent division through mitosis^11^. The cell cycle allows growth and proliferation of cells, development of tissues and whole organisms. It is also crucial in regulation of DNA damage repair, injury response and development of diseases such as cancer^12^. There is ever growing number of evidence supporting thesis, that deregulation of cell cycle machinery can induce disastrous changes in organism. For example, abnormal activation of cell cycle regulatory proteins lead to development of Alzheimer’s disease^13,14^. The cell cycle has been extensively studied for years which led to greater understanding of life of a cell and organism. As great understanding may be, there is still much to be discovered especially for chemical and physical processes taking place at the nanoscale.

Cell cycle investigation is possible thanks to the developed methods of synchronization of the cell cycle in cultured cells. They include either starvation or addition of external chemical agent^15^. One of inhibitors of the cell cycle – used for the purpose of synchronization – is aphidicolin. This tetracyclic diterpenoid isolated from *Cephalosporium aphidicola* and *Nigrospora oryzae* presents antimitotic and antiviral properties^16^. It also possesses the affinity towards DNA polymerase α, δ and ε. Through binding to those DNA polymerases it reversibly inhibits their function and stops the cell cycle at a G1/S border^17^. Despite not sharing structural homology with dCTP (deoxycytidine triphosphate) aphidicolin binds at this nucleotide-binding site^18^. It is expected, that aphidicolin inhibition does not affect DNA repair nor biosynthesis of RNA, and synchronized cells remain in physiological state and restart DNA replication shortly after removal of the compound^19^.

It this paper we present a study on changes of nanoviscosity of HeLa cells during cell cycle. It was expected, that significant structural changes in cells during biosynthesis or mitosis would have an impact on nanoviscosity, and further – diffusive motion of biomolecules. We measured the changes of relative diffusion coefficient of monomeric EGFP freely diffusing through cytoplasm of living human cells. EGFP was chosen as a noninvasive probe^20^. Diffusion coefficient of EGFP freely moving thorough cytoplasm of synchronized HeLa cells was observed using fluorescence correlation spectroscopy (FCS). FCS allowed precise measurements of mobility of EGFP in live cell cytoplasm as a function of time after removal of cell arresting agent. During experiments – to our surprise – cells maintained stable cytoplasmic nanoviscosity during whole cell cycle. This provides a premise, that some robust mechanism occurs in human cells, that provides stability of physiological nanoviscosity.

## Results

### Aphidicolin efficiently synchronizes HeLa cells

**Figure 1:**
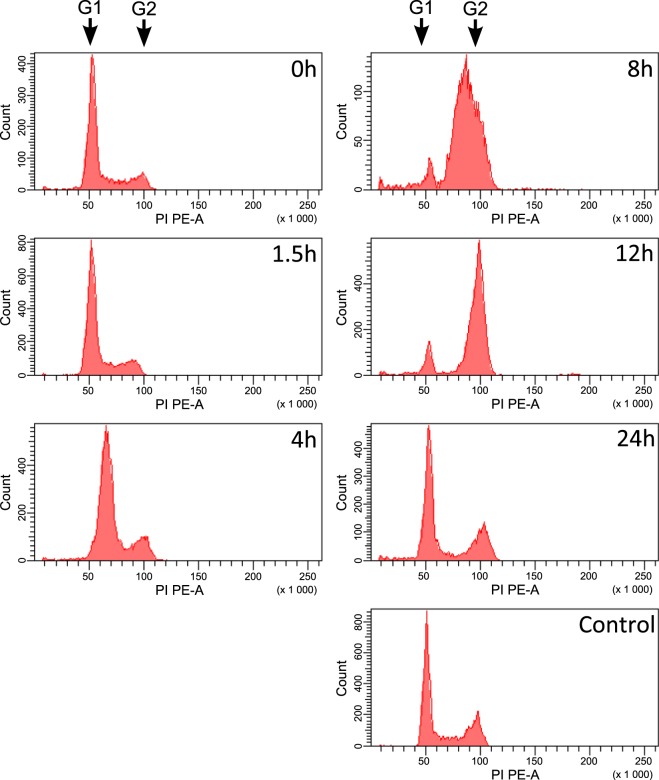
Aphidicolin efficiently synchronizes HeLa cells. HeLa cells were synchronized by 24 h incubation with aphidicolin. After aphidicolin removal, cells were collected at different time stamps (0, 1.5, 4, 8, 12 and 24 h). DNA content was measured by flow cytometry by tracing the fluorescence intensity of cells. Cells perform DNA replication (seen as doubling of fluorescent signal) in synchronized fashion. Control panel shows unsynchronized cells cultured the same way as other samples and collected simultaneously with cells after 24 h of aphidicolin block release. G1 and G2 with arrows denote peaks coming from cells in first and second gap phase respectively.

First, we tested if addition of aphidicolin indeed would allow robust synchronization of cell culture, by arresting the cell cycle in all cells in the culture. According to the literature data the action of aphidicolin can both arrest cells at the border of G1 and S phases but also could cause irreversible stop of the cell cycle^19,21^. For this experiment, we prepared synchronized cell cultures and subjected them to flow cytometry analysis. Basing on available literature data^22^ we picked few representative timestamps (0, 1.5, 4, 8, 12, and 24 hours after aphidicolin removal). In accordance with our predictions, the majority of cell population progressed in synchronized fashion. Figure 1. illustrates effectiveness of synchronization seen as transition of most prominent peak of fluorescence with time. Late S/G2 phase was reached around 12 hours after aphidicolin removal. After 24 hours cells return to their original, unsynchronized distribution (histogram matches closely that of the control). Our results are in agreement with similar experiments performed earlier^19^. Results of flow cytometry clearly show, that aphidicolin efficiently synchronizes HeLa cells and provides further cell cycle progress.

### Viscosity of cytoplasm of HeLa cells transiently increases during cell cycle

Knowing that chosen method of synchronization works as intended, we moved to measurements of diffusion of EGFP inside of HeLa cells. Cells constantly expressing EGFP were firstly synchronised and then examined using FCS after aphidicolin removal. Only viable cells, effectively attached to the surface could have been monitored using FCS, therefore accidental apoptotic events could have been omitted. Example FCS curve for EGFP in HeLa cells is presented in Supplementary Information file (Fig. SI.1). Intracellular and intercellular variances of FCS results were also analysed, and it was revealed, that population variability is more pronounced than intracellular heterogeneity (see Supplementary Information, Table SI.1).

**Figure 2:**
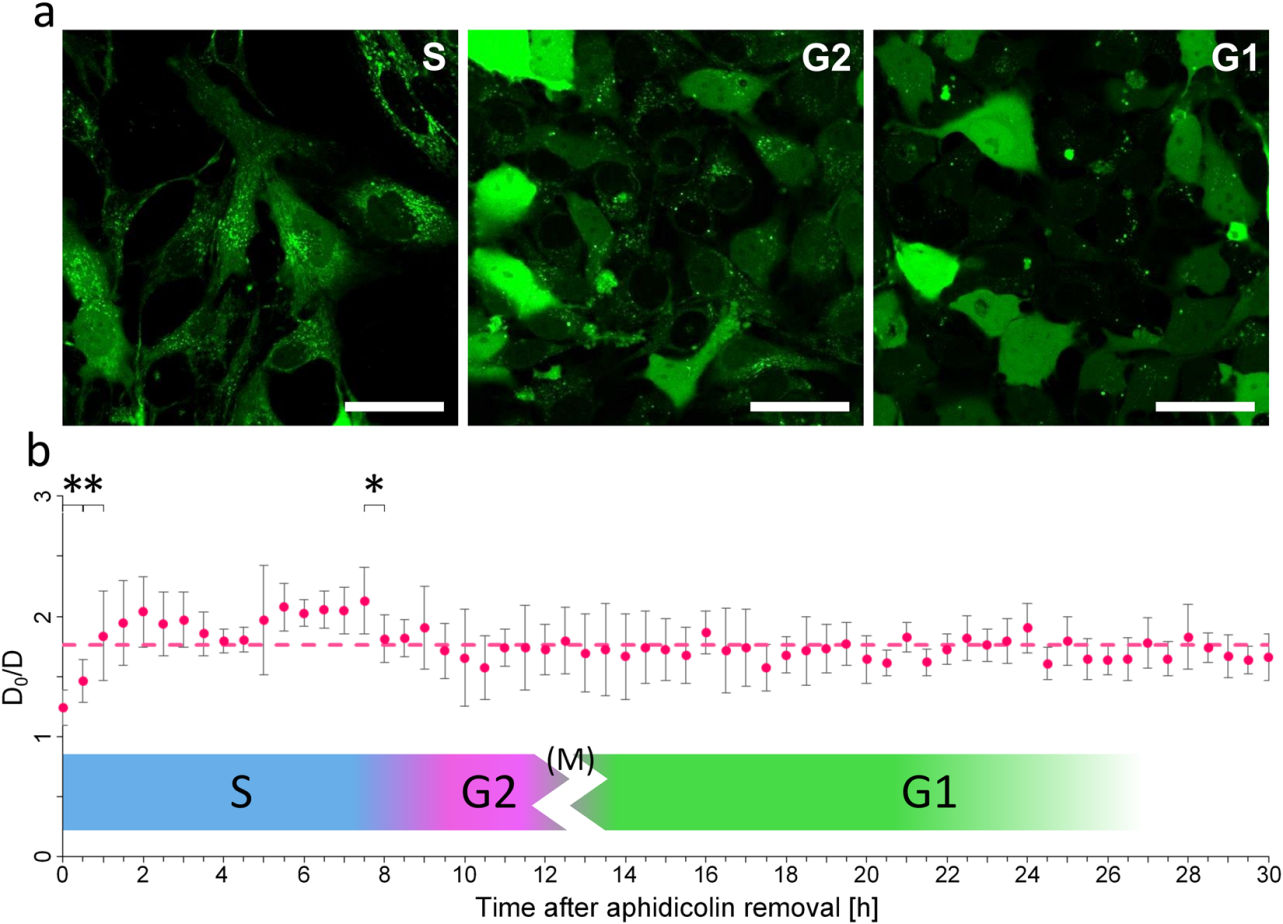
HeLa cells after removal of aphidicolin cell cycle block. (**a**) HeLa cells expressing EGFP were incubated with aphidicolin (5 µg/ml) for 24 hours. After removal of aphidicolin cells resumed cell cycle progression (starting from S phase). Pictures of cells at different stage of cell cycle were taken after 1.5, 10, and 14 hours after removal of aphidicolin. These represent S, G2 and G1 phase respectively. Scale bars correspond to 50 µm. (**b**) Changes in relative viscosity 1/D (with respect to water 1/D_0_) of EGFP freely diffusing in cytosol of HeLa cells. Diffusion coefficient in cytoplasm was measured by fluorescence correlation spectroscopy (FCS). Obtained data are presented as relative value of diffusion coefficient of EGFP in buffer (D_0_) to diffusion coefficient measured by FCS (D). Points represent mean value taken from at least 5 cells (for each cell 5 separate measurements lasting 60 s were taken). Error bars correspond to standard deviations. Dashed line represents mean value of all measurements (D_0_/D = 1.76). Asterisks (*) denote statistically significant changes between points. Unpaired Student’s t-test, P < 0,05.

After removal of aphidicolin cells first experience short lag (seen as viscosity lower that mean value from whole experiment) before they restart the DNA replication machinery. This observation was expected. Then, as the S phase commences, the relative viscosity (ratio of diffusion coefficient of EGFP in water (D_0_) to that measured in cytoplasm (D)) increases slightly. Value of EGFP diffusion in water was calculated from Stokes-Einstein equation^20^. Hydrodynamic radius of EGFP equal to 2.3 nm was calculated using HydroPro software^23^. As soon as the expected S phase finishes, relative viscosity drops and remains on constant level (D_0_/D ≈ 1.76) throughout the rest of cell cycle (Fig. 2b). Average value does not even change when mother and daughter cells are concerned^∗^. The only significant change in cytoplasmic viscosity was during S phase and it did not exceed 20% of a mean value. For comparison, cytoplasmic viscosity of cells exposed to osmotic shock of +100 mOsmol/L increases of 120% (see Supplementary Information, Fig. SI.2). It is also worth mentioning, that average value of relative diffusion coefficient measured here is almost 40% smaller than that obtained in unsynchronised cells (1.76 ± 0.28 in treated vs. 2.65 ± 0.6 in untreated cells). Data for unsynchronised cells (average of over 50 repetitions) were obtained in the same conditions i.e. aperture, temperature. This points to previously unforeseen, inhibitory effect of aphidicolin.

Possible contribution of EGFP to overall nanoviscosity of the cells was also analysed. Our calculations shown (see Supplementary Information: Table SI.2)^24^, that EGFP in cells used in experiments was ~0.01% of all cytosolic proteins in those cells. Thus, we state that this amount of EGFP can be neglected.

### Aphidicolin synchronization influences cell size

**Figure 3:**
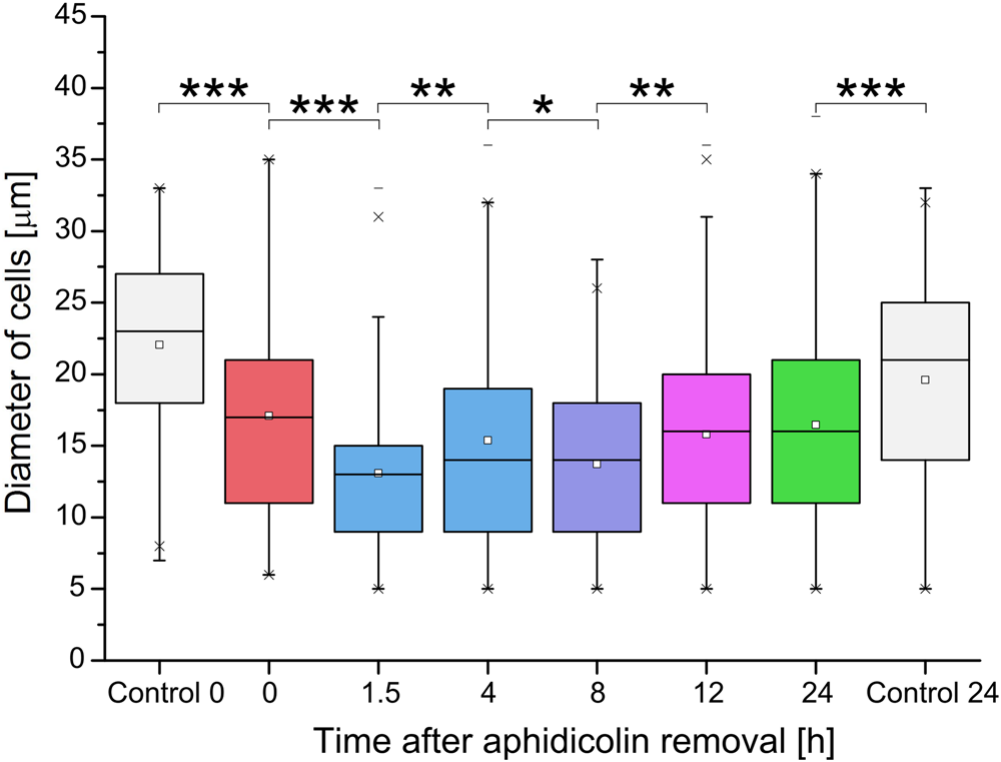
Aphidicolin synchronization influences cell size. HeLa cells were synchronized by incubation with aphidicolin (5 µg/ml) for 24 h. Cells were collected at different time stamps (0, 1.5, 4, 8, 12 and 24 h) after removal of synchronizing agent. Sizes of collected cells were measured with automatic cell counter. For analysis cells with diameter <5 µm were discarded as debris. Results are presented as box plots of six measurements from two separate runs of synchronization. The results are shown as box plots with small squares representing mean value, boxes – inter quantile range with median inside, and whiskers – 1^st^ and 4^th^ quadrille. Crosses (x) denote 99 and 1% of all the results and dashes (−) maximal and minimal values obtained. Statistical significance: unpaired Student’s t-test. Single asterisks (*) denote significant (P < 0,05), double (**) very significant (P < 0,005), and triple (***) extremely significant (P < 0,0005) statistical changes.

^1^

Having obtained surprising results we wanted to check how the overall cell size is connected with cell cycle and whether it was correlated with relative diffusion of EGFP. Synchronized cells were tested on the same timestamps as in experiments concerning action of the aphidicolin, i.e. 0, 1.5, 4, 8, 12, and 24 hours after agent removal. The results are presented on Fig. 3. The first observation is that incubation of cells with aphidicolin, causes overall decrease of size of cells. Despite supposedly not having any action on cell metabolism (apart from cell cycle arrest), this synchronizing agent seems to influence also other systems. Cells do not recover from this change even after 24 hours of undisturbed growth. Also, the slight, statistically significant increase of cell size during measurements can be observed. This further supports hypothesis, that aphidicolin synchronisation has prominent effect on cell physiological state. This effect must be indeed powerful, since 30 hours (so at least one generation) of undisturbed growth do not completely alleviate it. According to Kung *et al*.^25^ human cells protect themselves from negative effects of cell cycle arrest by, among others, inhibition of protein synthesis. Undesirable, high levels of toxic proteins could prove disastrous to well-being of a cell. Bearing that in mind we hypothesize, that, indirectly, aphidicolin exposure can cause reduction in protein synthesis. Therefore, overall decrease of cell size and, possibly, viscosity occurs.

## Discussion

We have measured the viscosity of the cytoplasm seen as changes of relative diffusion coefficient of EGFP during the cell cycle of HeLa cells. Although few other groups also sought insight into this issue, their works aimed to elucidate different phenomena. Basing on vast literature data concerning cell cycle we hypothesised that enormous structural changes that take place inside of living cell which prepares for division would have impact on viscosity of the cytoplasm. To our surprise, results we got suggested different scenario, one in with cytoplasm viscosity remains on stable, physiological level. Liang *et al*. also studied nucleoplasmic and cytoplasmic viscosity of HeLa cells during cell cycle^10^. In their work they focused their attention only on three timestamps of cell cycle representing major interphase phases: G1, S and G2. As did we, they noticed significant difference between cells synchronized in S phase and outside of it. However, their results suggest that cytoplasm is less viscous during DNA synthesis. One could argue that this disagreement could originate from method of synchronisation. For each timestamp of cell cycle they used different method/agent, each acting on different level of cell architecture. This could cause distinctive modifications of cell metabolism. As we showed in present work, synchronization of cells using aphidicolin greatly impacts their physiology. We proved, that not only the cell cycle progression is influenced, but also overall size of cells and their viscosity is altered. Our results also show, that this perturbation of cell physiology poses such an impact, that cells do not recover even after hours of undisturbed growth. At the end of experiment, 30 h after removal of cell cycle arrest, viscosity and size of cells still was significantly lower than in those of untreated controls. We therefore suspect that, cells exposed to different procedures may have significantly altered characteristics and should not be compared as equals.

Our data show, that EGFP diffusion in cells (and therefore also the viscosity of cytoplasm) before and after the division do not differ. Alas, precise identification of the time of division of cell was not possible. Due to limitations of the technique, no cells undergoing mitosis were studied. Therefore, we were not able to monitor possible transient change of viscosity of cytoplasm when the nuclear envelope disappears.

Basing on data obtained by Pedrali-Noy *et al*.^19^ we assumed that when aphidicolin was present in the media cells would function normally, waiting for the DNA to be replicated. Yet, in their work Kung and his colleagues^26^, shows that in HeLa cells the inhibition of DNA synthesis causes down-regulation of protein synthesis and arrest of cellular growth. This helps cells to limit the toxic effect of abnormal concentrations of regulatory proteins. It becomes even more likely, when cytotoxic effects of synchronization agents are taken into consideration. Cell cycle arrest ‘toxicity’ comes not from direct action of chemicals *per se* but rather derive from change of ratio of cell size to DNA content^25^. As example of similar, extraordinary control mechanism other phenomena present in cell can be used. Posakony *et al*. proved the fraction of cytoplasm volume occupied by mitochondria in undisturbed cells remains constant throughout whole cell cycle^27^. Taken together all those observations suggest that all cellular processes which have an impact on cellular volume are somehow orchestrated.

Concentration of macromolecules in cytoplasm reaches up to 400 mg/ml and most processes taking place inside the cytoplasm are diffusion dependent^28,29^. Noticing that, it is plausible that even seemingly small deviations from optimal level of crowding could prove disastrous for living cells. In fact, it was previously shown that kinesin-1, regarded as one of more robust transport protein, is indeed highly susceptible to changes of nanoviscosity^5^. But not only the active transport can fall victim to deregulation of cytosol viscosity. Diffusion limited reactions are even more susceptible to changes in surrounding environment. Few works have shown the extend of this effect^2,30^. Hence, it is not surprising that robust mechanism of coping with structural changes taking place in cell cycle exists. Still, presence of such mechanism remains a hypothesis and needs further investigation. Nevertheless, cells certainly are able to avoid drastic disturbances of viscosity of cytoplasm. In this way, all metabolic processes needed for sustaining well-being of a cell remain in check.

Despite lack of previous literature evidence, the impact of aphidicolin synchronization seems prominent. The action of aphidicolin is restricted only to inhibition of DNA polymerases, yet, in consequence, overall condition of the synchronized cells is influenced. It is known, that this agent binds to dCTP (deoxycytidine triphosphate) binding site and causes distortions in polymerase structure. Also, it creates sterical hindrance for dCTP, thus preventing incorporation of this nucleotide^18^. It was previously shown, that replication and transcription of DNA have to be carefully spatially controlled because collisions of those two processes lead to perturbations of cell function^31,32^. During aphidicolin block no new mRNA is created, so translation and protein production also cease. Thus, one should be careful when applying any cell cycle blocks. Without prior tests, the possible impact of such interferences is unknown. This places work of Liang *et al*. in question^10^. In their studies of cell cycle (in principle similar to present investigation) they applied three different methods of cell arrest (mimosine for G1, thymidine block G1/S boundary and colchicine for G2), yet failed to determine their impact. Application of single chemical agent (and its molecular target) seems to be less prone to unexpected difficulties.

To conclude, in this study we investigated changes of mobility of EGFP inside of the cytoplasm of the HeLa cells during cell cycle. Applying fluorescence correlation spectroscopy, we obtained only slight, yet significant increase of nanoviscosity during synthesis phase, whilst for the rest of the cell cycle the nanoviscosity remained stable. Our results suggest, that viscosity of the cytoplasm is conserved throughout majority of the life of a cell. Despite huge changes connected with existence of cell cycle (cell size, amount and composition of proteins, DNA duplication), no alteration of mean physiological threshold was observed. This stability suggest, that nanoviscosity – which is a key determinant for diffusion-limited reactions – may play an important role for cell metabolism and fate. Our research opens a field for new questions about nanoviscosity of the cells and its possible impact of on cellular functions.

## Methods

### Cell culture

HeLa-EGFP inducible line (EGFP expressed under tetracycline inducible suCMV promoter) was purchased from AMS Biotechnology (http://www.gentarget.com/product/inducible-gfp-hela-stable-cell-line). Cells were cultured with Dulbecco’s modified Eagle’s medium (Institute of Immunology and Experimental Technology, Poland) supplemented with 10% heat-inactivated fetal bovine serum, L-glutamine (2 mM), penicillin (100 mg/ml), streptomycin (100 mg/ml) and 1% non-essential amino acids (all from Sigma-Aldrich, USA). Cells were maintained at 37 °C in a 5% CO_2_ humidified atmosphere. For synchronization and subsequent fluorescence correlation spectroscopy analysis cells were grown on 35 mm thin glass bottom dishes (Eppendorf, Germany) to approximately 50% of confluence. For flow cytometry analysis cells were grown on six-well plates (Greiner Bio-One, Austria) instead. At no point of culture the expression of EGFP was induced, for FCS measurements (requirement of ~1 fluorescent molecule per focal volume) amount of EGFP produced solely as a result of suCMV promoter leakage was sufficient.

### Aphidicolin synchronization

Ready-made aphidicolin solution (1 mg/ml) was purchased from Sigma-Aldrich (USA). Cells were seeded on either 35 mm glass bottom dishes or on six-well plates and allowed to adhere for 24 hours before aphidicolin synchronization (2 ml of medium in total). After that, 10 µl of aphidicolin solution was added and allowed to act for 24 h. Then medium containing aphidicolin was removed and replaced with 2 ml of aphidicolin-free medium. Then, culture dishes were left in incubator for appropriate amount of time. Cells prepared in such way were subjected to analyses.

### Fluorescence correlation spectroscopy

Cells cultured on glass bottom dishes were first washed with 1 ml of phosphate buffer saline (PBS). Then cells were covered with 2 ml of PBS for measurements. Synchronized cells were prepared as described in previous section. Control cells were cultured on glass bottom dishes overnight just before the measurements. FCS measurements were performed using commercially available setup based on confocal microscope (Nikon Eclipse TE2000U, Nikon, Japan) coupled with Pico Harp 300 TCSPC module (PicoQuant, Germany). All measurements were performed in a climate chamber (Okolab, Italy) providing temperature control (36 ± 0.5 °C) and atmosphere of required composition and humidity. Pulse diode laser (PicoQuant LDH-D-C-485; 485 nm) driven by Sepia II module (both from PicoQuant, Germany) was used as a light source. Power of the laser was kept in the range of 5–10 μW, which was low enough to avoid excessive EGFP bleaching during cell studies. Probes were observed and measured through 60 × (N.A. 1.2) objective with water immersion. Fluorescent signal for FCS was collected by single photon avalanche diodes (MPD and PerkinElmer, USA).

Each experiment with cells was preceded with a calibration step. Rhodamine 110 (Sigma-Aldrich, USA) in 2.5%_weight_ glucose in PBS was used for calibration and correction collar adjustment^20^. Diffusion coefficient of Rhodamine 110 in the calibration conditions was 560 µm^2^/s and this value was used for calculation of a size of a focal volume. On average short axis of the focal volume (ω_0_) was equal to 0.196 ± 0.009 µm, structure parameter (κ) to 6.4 ± 0.8, and effective volume (V_eff_) to 0.27 ± 0.04 fl.

Position of a cell was adjusted using confocal microscope. Confocal focus was positioned in a cytoplasmic area of a cell. SympPhoTime 64 software (PicoQuant, Germany) was used for time traces acquisition and autocorrelation curves calculation. Fluorescence lifetime filtering was applied to improve the signal to noise ratio and eliminate the afterpulsing effect from the autocorrelation curves. Data was acquired in 2 separate runs, 5 measurements per cell, each lasting 60 s. Autocorrelation function fitting according to 1-component free diffusion model was performed using QuickFit 3.0 software (Division Biophysics of Macromolecules, German Cancer Research Center, Germany) with FCS Correlation Curve Fitting Plugin, function FCS: 3D Normal Diffusion, Levenberg-Marquard fit (without box constraints).

### Point acquisition and statistical analysis

For construction of plot depicting changes of relative diffusion coefficient after aphidicolin removal in live cells following procedure was used. For each cell the average value of diffusion coefficient of EGFP was obtained from 5 consecutive measurements. The time of start of measurements (related to removal of aphidicolin) was noted. All cells were then grouped into subgroups and averaged (point 0 includes all measurement taken from 0 to 30 min after removal of cell cycle block, point 30–30 to 60 min, etc.). Thus, each point on plot represents average value obtained from 5–10 cells.

Data for unsynchronized cells were obtained in analogous way. For determination of EGFP diffusion coefficient in cytoplasm data from over 50 cells were pooled.

### Flow cytometry

Cytometric analysis was performed by National Medicine Institute (Warsaw, Poland) as a commercial order. For cytometric analysis cells were first cultivated on 35 µm dishes as described earlier. Grown cells were then additionally prepared as follows. Culture media was collected from culture then cells were washed with 0.5 ml of phosphate buffer saline (PBS). PBS was also collected and 200 µl of 0.25% Trypsin-EDTA solution (Sigma-Aldrich) was added. Cells were then left for 1 min incubation. Trypsin action was then quenched with 1 ml of previously collected media. Cells were detached from surface of dish and joined with previously collected fluids. Samples were then centrifuged (3500 rpm, 5 min) and supernatant was discarded. Precipitate was suspended in 1 ml of fresh PBS and centrifuged again. Cells were then suspended in 500 µl of fresh PBS. Afterwards, using Pasteur pipette, suspension was vigorously injected under the surface of ethanol (5 ml; 70%_volume_) previously stored in freezer (−20 °C). Cells were collected at six different timestamps, 0, 1.5, 4, 8, 12 and 24 hours after aphidicolin removal. Each cell collection was repeated twice. Also three repetitions of unsynchronized cultures were collected. There cells were stained with appropriate solution (Trition X-100, propidium iodide, RNase) and analyzed by BD FACSCanto II apparatus (BD Biosciences, USA). Data analysis was performed employing FCS Express 4 (De Novo Software, USA).

### Cell size determination

Cells were cultivated on 35 µm dishes as described in flow cytometry protocol. For size analysis 10 µm of harvested cells suspension was mixed with 10 µm of trypan blue solution (0.4%; Thermo Fisher Scientific, USA). Then 10 µm of such prepared cell suspension was transferred onto cell counting slide and measured on Countess™ II FL Automated Cell Counter (Thermo Fisher Scientific, USA): cells were imaged and diameter of each cell was measured. The size histograms were extracted and average cell size calculated. Cells with diameter <5 µm were excluded from calculations as possible debris. For each time stamp (0, 1.5, 4, 8, 12 and 24 hours after aphidicolin removal) measurement was repeated 3 times for two separate synchronization runs. Overall average value was obtained by pooling all results.

### Image acquisition and analysis

Image acquisition was performed concurrently with FCS measurements, using the same equipment. Pictures were taken using fluorescent lifetime module (FLIM) also present as a part of SymPhoTime software. Further picture analysis was performed applying ImageJ software.

## Supplementary information

Supplementary Information

## Footnotes

1 As mother cells we understand cells pre-division ~12 h after aphidicolin removal, and by daughter cells observed few hours later.
