# Supplementary material for: Stability of cytoplasmic nanoviscosity during cell cycle of HeLa cells synchronized with Aphidicolin
Source: Sci Rep. 2019 Nov 11;9:16486. doi: 10.1038/s41598-019-52758-6 (PMC6848169; doi:10.1038/s41598-019-52758-6)
Supplement: Supplementary file 1 — Supplementary Information [file 41598_2019_52758_MOESM1_ESM.pdf]

Supplementary information

for

Stability of cytoplasmic nanoviscosity during cell cycle of HeLa cells synchronized with Aphidicolin

Krzysztof Szczepanski,[1] Karina Kwapiszewska,[1] & Robert Holyst[1*]

[1]Institute of Physical Chemistry, Polish Academy of Sciences, Kasprzaka 44/52, 01-224 Warsaw, Poland;

**Corresponding author: holyst@ichf.edu.pl*

FCS of EGFP in cytoplasm of HeLa cells

EGFP freely diffuses in cytoplasm of HeLa cells (see reference 20), and thus FCS autocorrelation curve recorded for its motion can be fitted with one-component 3D diffusion model with triplets (Equation SI.1):

$$G(\tau) = \frac{1}{N}\left(\frac{1-p_{trip}+p_{trip}e^{-\tau/\tau_{trip}}}{1-p_{trip}}\right)\frac{1}{1+\frac{\tau}{\tau_D}}\,\frac{1}{\sqrt{1+\frac{1}{\kappa^2}\frac{\tau}{\tau_D}}}, \qquad \text{(SI.1)}$$

where N stands for the average number of fluorescent probes inside the focal volume, τ_D is the average time of diffusion of a probe across the focal volume, κ is the aspect ratio of the focal volume (measured during calibration), p_{trip} is fraction of triplet states and τ_{trip} is a decay time of a triplet state.

Each autocorrelation curve was fitted with N, τ_D, p_{trip} and τ_{trip} as free parameters. Example fit is presented in Figure SI.1.

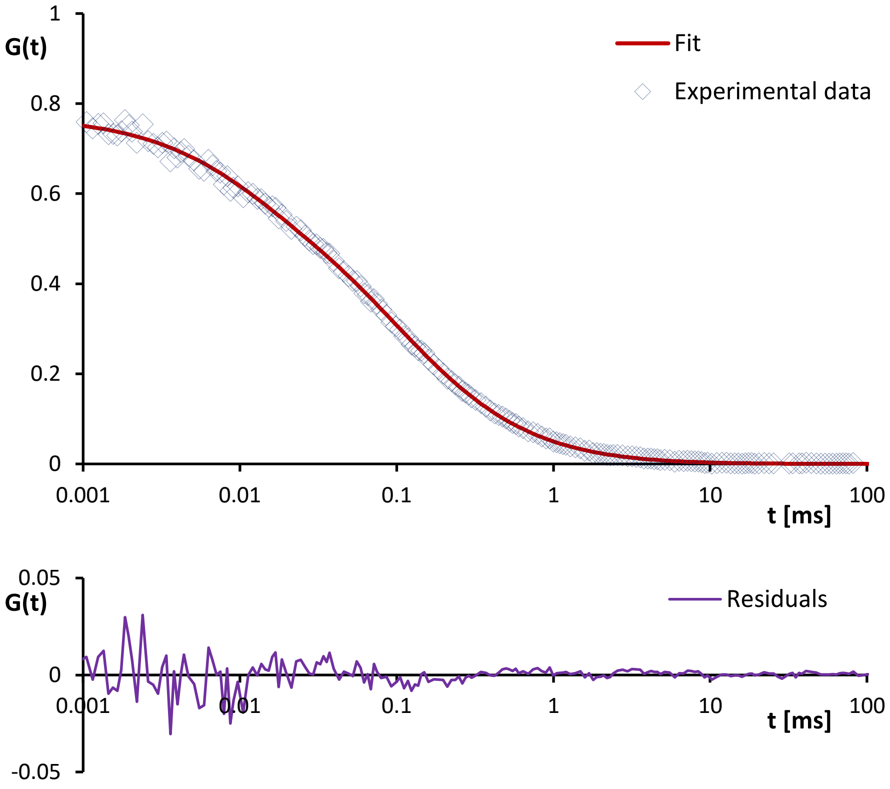

Figure SI.1. Autocorrelation curve for EGFP in cytoplasm of a HeLa cell after Aphidicolin synchronization. Experimental data fitted well with a one-component 3D free diffusion model with triplets.

Intracellular and intercellular variance of results

Control experiment was performed to compare inter- and intracellular variances of diffusion coefficients of EGFP measured by FCS in the cytoplasm of HeLa cells. To reveal cytoplasmic heterogeneity, FCS was performed at 8 different spots, picked randomly in the cytoplasmic area of the cell. For each cell, variance and average of the results was calculated. Variance for diffusion coefficients measured for different cells varied between 12 and 52% of a mean values for given cells (average variance was 42%). For comparison, variance of average diffusion coefficients of the cells considered in this experiment was 128%.

Variance of the diffusion coefficients for the 0.5h time windows considered in the cell cycle experiment varied between 17 and 670%, with the average of 129%. The data was presented in Table SI.1.

Table SI.1. Variance of cytoplasmic viscosity (intracellular and intercellular).

		**Intracellular**	**Intercellular** *control experiment*	**Intercellular** *cell cycle experiment*
variance	Min	12%	-	17%
	Max	52%	-	670%
	Average	42%	128%	129%

Changes in cytoplasmic viscosity upon osmotic shock

Cells were exposed to osmotic shock to measure levels of cytoplasmic viscosity that are possible to achieve in HeLa cells for EGFP protein. For this purpose Influx hypertonic cell culture medium was used (Influx kit, Sigma-Algrich). The stock hypertonic medium had osmolarity of 900 mOsmol/L, while basic cell culture medium is 300 mOsmol/L. A series of dilutions of the stock solution in cell culture medium resulted in media of 400 mOsmol/L, 500 mOsmol/L, 600 mOsmol/L, 700 mOsmol/L and 800 mOsmol/L. EGFP diffusion coefficients could have been measured with FCS for the osmolarity of up to 600 mOsmol/L. The results are presented in Figure SI.2.

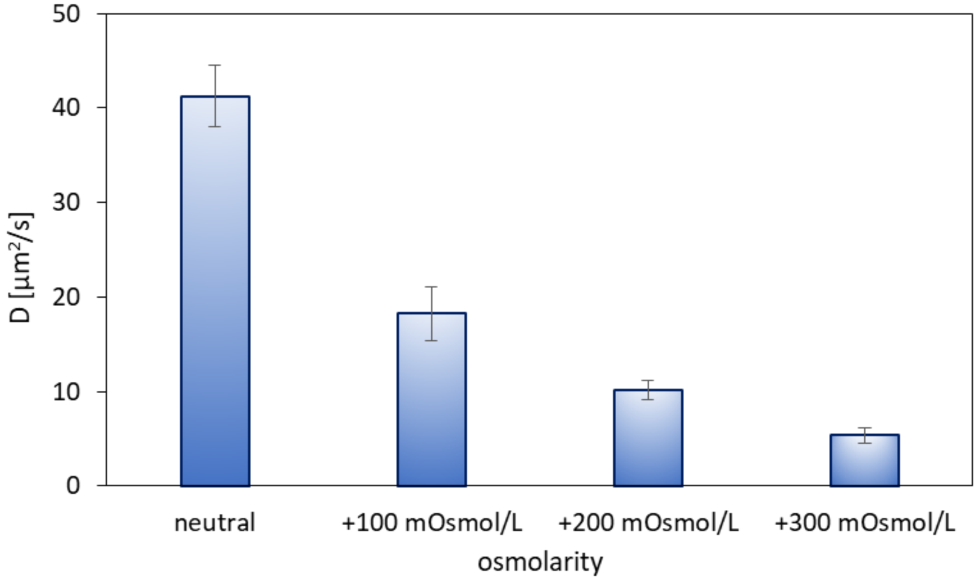

Figure SI.2. Diffusion coefficients of EGFP in the cytoplasm of HeLa cells exposed to media of different osmolarity.

According to our results, cytoplasmic viscosity increase was 120%, 300% and 670% for osmolarity increase of 100, 200 and 300 mOsmol/L respectively.

Contribution of EGFP to overall cytoplasmic crowding

The cell line used in experiments was HeLa-EGFP inducible line, where EGFP is expressed under tetracycline inducible suCMV promoter. For FCS measurements it is required to have ~1 fluorescent molecule per focal volume. Thus, amount of EGFP produced solely as a result of suCMV promoter leakage was sufficient for our experiments and no induction was applied to enhance EGFP expression.

All FCS measurements were performed in the regime of 1-10 EGFP molecules per focal volume. It corresponds to total number of ~70 000 EGFP copies per cell. According to reference 24 total number of copies of proteins natively present in cytosol is ~640 000. Thus, EGFP expressed additionally is 0.01% of total number of cytosolic proteins. We believe, that contribution of such a rare crowder can be neglected in overall crowding.

Data used for calculation are presented in Table SI.2.

Table SI.2

Quantity	Value	
Average focal volume	0.2	fL
Number of GFP copies per focal volume	10.0	
Average cell diameter	15.0	µm
Average cell volume	1.8	pL
Average cytosol volume	1.4	pL
Number of GFP copies in measured cell	70 313.0	
Number of native protein copies	639 820 582.0	
